# Infirmary Medical Superintendents' Recommendations

**Published:** 1918-08-10

**Authors:** 


### THE REFORM OF THE POOR LAW.

### Infirmary Medical Superintendents' Recommendations.

Attached to the Report on Poor-Law Reform forwarded to the Minister of Reconstruction by the Infirmary Medical Superintendents' Society are three appendices. The first appendix deals with the district hospitals. The Society points out that a Poor-Law infirmary has to provide beds not only for the various kinds of illness taken in by voluntary hospitals, but also for diseases which voluntary hospitals refuse, such as erysipelas, phthisis, measles, whooping-cough, chicken-pox, and all kinds of chronic disease. The provision made by different Boards of Guardians varies very much, but in no instance is it completely satisfactory. As shown in the main Report a new authority would overcome many of the difficulties by establishing special hospitals. The local hospital would, however, still have to provide for a very varied assortment of patients.

### The District Hospital as a Teaching Centre.

The Society estimates that a district hospital would have to have one bed for every three hundred of the population, and that each hospital would need a number of small isolation and special wards. There should be two operating-rooms, one for septic and one for aseptic cases, an efficient *x*-ray apparatus, and a laboratory for routine pathological and bacteriological work. The clinical material should be available for the training of medical students and for post-graduate teaching, and arrangements should be made for the compilation of medical statistics. The medical superintendent should be responsible for the administration of all the local medical institutions provided by the authority within his district and for the supervision of the domiciliary medical treatment undertaken by it. He should be assisted by two resident medical officers, who should be paid at least £500 a year and provided with married quarters. One of them should be his deputy, and should be responsible for part of the administrative work. Junior assistant medical officers, who need not necessarily be whole-time or resident, would be required in proportion to the size of the hospital.

### A Uniform Examination for Probationers.

The second appendix deals with the training of probationer nurses. The Society points out that the large infirmaries, owing to the number of bed-ridden patients and of acute medical and surgical cases they contain, possess the very best material for the thorough training of probationer nurses. Many of the smaller infirmaries are not fitted for the purpose, because the amount of surgical work done in them and the number of acute cases admitted are too small. It is recommended that a minimum curriculum of training be drawn up, and that all the nurses in the service be required to pass the same official examinations before being granted certificates of training. There should be no other interference with the individual training-school. Any system by which the probationer would receive part of her training in one school and the remainder in another is deprecated. If infirmaries are organised so that some receive acute and others chronic cases, it is suggested that each infirmary for acute cases should have attached to it one for chronic cases under the same medical and nursing administration so as to form one training-school.

### Co-ordinating Home and Institutional Assistance.

The third appendix deals with Home Medical Assistance. It is recommended that this should be under the supervision of the medical superintendent of the district hospital, and be carried out mainly by medical officers attached to it. In this way the institutional and home medical assistance would be co-ordinated, cases for hospital treatment would be selected with a full knowledge both of the home conditions and of the hospital accommodation available, and after-treatment in the home would be more efficient.

